# Orientation-Independent Human Activity Recognition Using Complementary Radio Frequency Sensing

**DOI:** 10.3390/s23135810

**Published:** 2023-06-22

**Authors:** Muhammad Muaaz, Sahil Waqar, Matthias Pätzold

**Affiliations:** Faculty of Engineering and Science, University of Agder, 4898 Grimstad, Norway; sahil.waqar@uia.no (S.W.);

**Keywords:** activity recognition, data fusion, distributed mmWave MIMO radar, fall detection, feature extraction, micro-Doppler signature, mean Doppler shift, support vector machine

## Abstract

RF sensing offers an unobtrusive, user-friendly, and privacy-preserving method for detecting accidental falls and recognizing human activities. Contemporary RF-based HAR systems generally employ a single monostatic radar to recognize human activities. However, a single monostatic radar cannot detect the motion of a target, e.g., a moving person, orthogonal to the boresight axis of the radar. Owing to this inherent physical limitation, a single monostatic radar fails to efficiently recognize orientation-independent human activities. In this work, we present a complementary RF sensing approach that overcomes the limitation of existing single monostatic radar-based HAR systems to robustly recognize orientation-independent human activities and falls. Our approach used a distributed mmWave MIMO radar system that was set up as two separate monostatic radars placed orthogonal to each other in an indoor environment. These two radars illuminated the moving person from two different aspect angles and consequently produced two time-variant micro-Doppler signatures. We first computed the mean Doppler shifts (MDSs) from the micro-Doppler signatures and then extracted statistical and time- and frequency-domain features. We adopted feature-level fusion techniques to fuse the extracted features and a support vector machine to classify orientation-independent human activities. To evaluate our approach, we used an orientation-independent human activity dataset, which was collected from six volunteers. The dataset consisted of more than 1350 activity trials of five different activities that were performed in different orientations. The proposed complementary RF sensing approach achieved an overall classification accuracy ranging from 98.31 to 98.54%. It overcame the inherent limitations of a conventional single monostatic radar-based HAR and outperformed it by 6%.

## 1. Introduction

The world is undergoing a demographic shift, with the elderly population rapidly increasing in almost every country. By 2050, nearly 2.1 billion people will be over the age of 60 [1]. People of an older age are generally considered frail and often characterized by geriatric syndromes. Therefore, there is a growing need to develop new ambient intelligent spaces and AAL technologies to ensure that the elderly can safely age in their homes for as long as possible. Apart from making elderly people’s lives easier, these technologies also make them self-supporting in performing their ADLs without seeking help from other people and thus alleviating the high costs associated with elderly care. To facilitate self-dependency, AAL systems rely on decision support systems to proactively ascertain the context through the user’s behavior, activities, and interactions with the environment. Fall detection and prevention is another key aspect of AAL systems, because frail elderly people are at an increased risk of falling and injuring themselves. Therefore, HAR and fall detection systems are the essential building blocks of any robust AAL system. In addition, robust HAR is important for properly monitoring and quantifying physical activities, which can contribute to various smart-health [2] and wellbeing applications [3,4].

The existing HAR systems can be classified into three main categories: wearable sensor-based, vision-based, and RF-based systems. Wearable sensor-based HAR systems generally use different types of on-body sensors such as PLIMUs [5], surface electromyography (sEMG) electrodes [6], and/or pressure sensors [7] to collect dynamic body data, which are then used to recognize human activities. PLIMUs are the most widely used on-body sensors for wearable sensor-based HAR systems, because they are small in size, cost-effective, lightweight, and readily available through smartphones or smartwatches. There also exist other types of on-body sensors, which are usually embedded in wearable textiles [7,8]. In addition, wearable HAR systems are known to be quite accurate. Despite all these strengths, wearable sensor-based HAR systems suffer from several drawbacks that limit their practicality, affect their ease of use, and reduce their data quality [9]. For example, they are invasive, and users must carry on-body sensors at all times for continuous HAR. Moreover, the performance of wearable sensor-based HAR systems is highly compromised if the sensors are not placed correctly on the human body according to the system provider’s guidelines. Vision-based HAR systems, on the other hand, use cameras and computer vision techniques to recognize human activities [10,11,12]. Such systems generally perform very well and, in some scenarios, they can efficiently recognize and monitor the activities of multiple persons [13]. That being said, the performance of vision-based HAR systems is highly affected by the environmental settings, such as the lighting and cluttered or dynamic background conditions [10,12]. Moreover, vision sensors are understood as a threat to user privacy due to security issues that often stem from the operational design of vision sensors [14].

RF-based HAR systems use RF devices to emit electromagnetic waves and exploit the characteristics of the received signals reflected by the user’s body to recognize their activities. In recent years, RF-based approaches for HAR have attracted a lot of research interest due to their advantages over wearable sensor- or vision-based approaches for HAR. For example, they offer high environmental adaptability and can recognize human activities without risking privacy and comfort [15]. Wi-Fi [2,16,17,18,19] and mmWave radar [15,20,21,22,23,24,25] are the most widely used sensing technologies within the context of RF-based HAR systems. Note that the term mmWave radar is generally used to refer to radars that use short-wavelength electromagnetic waves. Wi-Fi, as an RF sensing technology, offers low sensitivity and low spatial resolution compared to mmWave radar technology. Consequently, the performance of Wi-Fi-based HAR systems is generally lower than that of mmWave radar-based HAR systems. Therefore, mmWave radars have become the de facto choice for RF-based HAR research in recent years. Like wearable and vision-based HAR systems, the performance of mmWave radar-based HAR system also suffers from various environmental factors and hardware limitations. Usually, mmWave radar-based HAR systems use the output of the radar (i.e., the micro-Doppler signature or 3D point cloud) that is computed from the received RF signals to classify human activities. These outputs are highly sensitive to the aspect angle between the boresight of the radar and the person’s direction of motion. This means that the micro-Doppler signature of an activity changes as the aspect angle between the boresight of the radar and the person’s direction of motion changes. As a result, the micro-Doppler signatures of an activity performed at 0° and 90° aspect angles are quite different from each other and resolve into highly uncorrelated intraclass features from a machine (deep) learning perspective. Therefore, the current state-of-the-art mmWave radar-based HAR systems that use a single monostatic radar struggle to recognize orientation-independent human activities [26,27].

In this work, we propose a complementary RF-sensing approach to realize orientation-independent HAR tasks. Our approach uses a distributed MIMO radar system that was configured to comprise of two separate monostatic radars operating in a TDMA scheme. These radars are placed orthogonal to each other in the environment to illuminate the target from 0° and 90° aspect angles. Each radar provides its own micro-Doppler signature, which complement each other such that the micro-Doppler signature of an activity obtained by combining the outputs of both radars always remained sufficiently distinctive, regardless of the orientation in which the activity was performed. Thus, our proposed solution with the following contributions advances the state of the art towards designing a high-performance orientation-independent RF-based HAR system:

- We propose a complementary RF sensing approach to overcome the limitations of contemporary mmWave monostatic radar-based HAR systems and robustly recognize orientation-independent human activities.
- We systematically evaluate the performance of the proposed approach for recognizing orientation-independent human activities.
- We extract several statistical and time- and frequency-domain features from the outputs of the radars that allowed the SVM to robustly classify human activities.
- We show that the fusion of the features obtained from the outputs of orthogonally placed complementary radars enabled our HAR system to classify orientation-independent human activities with a higher accuracy than the current state-of-the-art models.

The rest of the paper begins by presenting the related work in Section 2. Section 3 introduces the proposed orientation-independent HAR system. Section 4 provides an insight into the distributed mmWave MIMO radar system and the processing of the received signals. The details of the experimental setup and methodology for collecting the orientation-independent data are presented in Section 5. Section 6 systematically describes our approach for processing the collected orientation-independent human activity data. Section 7 presents the supervised learning setup, feature extraction, feature fusion, and in-depth analysis and discussion of the results. Finally, Section 8 concludes the paper.

## 2. Related Work

In recent years, the use of mmWave radar as an RF sensing technology has increased in various human-centric applications, such as HAR [15,20,21,22,24,25]; gesture recognition [28,29,30]; gait recognition [31,32]; human step counting [4]; fall detection [23,33]; and sign language gesture recognition [34]. In general, the tasks of recognizing human activities, (sign language) gestures, and detecting accidental falls using mmWave radar follow similar steps. At first, the reflected RF signals collected using the mmWave radar are processed to compute different types of radar outputs (e.g., the Doppler profile, range profile, and 3D point cloud), which are then used to classify human activities using learning techniques.

In [20], micro-Doppler spectrogram images and a CNN were used to classify targets and human activities including “running, walking, walking while holding a stick, crawling, boxing while moving forward, boxing while standing in place, and sitting still”. The proposed approach was able to classify human activities with 90.9% accuracy and distinguish humans from dogs, horses, and cars with 97.6% accuracy. In [21], the authors experimented with LSTM networks and their bidirectional variants, known as Bi-LSTM networks, to classify human activities using micro-Doppler and time-range profiles. The Bi-LSTM network together with the micro-Doppler information was able to classify six human activities with over 90% accuracy, whereas the time-range profile achieved nearly 76% accuracy. A novel 3D point-cloud-based non-invasive HAR system was presented in [22] that used an enhanced voxelization approach to create spatial-temporal point clouds and a dual-view CNN to classify the activities. The proposed approach could classify seven activities with 98% accuracy and detect falls with 97.61% accuracy. Another work [15] fused the features extracted from the point cloud and the range-Doppler information to classify six different human activities, i.e., “boxing, jumping, squatting, walking in place, high knee lifting, and circling in place”. They used a CNN-LSTM network to extract features from 3D point cloud data and a CNN network to extract features from the range-Doppler profile. Finally, both feature maps were fed into a fusion network consisting of a concatenation layer and fully connected layers that first concatenated the feature maps of both networks and then classified the human activities. The proposed fusion network classified the six aforementioned human activities with an overall recognition accuracy of 97.26%. Moreover, the findings of this work suggested that the classification accuracy for human activities based on fused feature maps was slightly better than the classification accuracy achieved using any of the isolated feature maps.

The performance of RF-based HAR systems depends on various environmental factors that have been superficially addressed in the literature, such as the effect of different environments and the aspect angle between the radar and the person performing the activities. In [25], the performance of an RF-based HAR system was evaluated across environments. The results of this work suggested that the micro-Doppler signatures of human activities do not change significantly in different environments. Moreover, in the literature, the performance of RF-based HAR systems has usually been assessed with respect to a 0° aspect angle for the target (i.e., the person performing the activities), which yields the best recognition accuracy. From a realistic perspective, a user should be able to perform activities freely in any direction and at any location within the radar’s range. However, activities performed at different aspect angles produce different micro-Doppler signatures [35], which significantly downgrades the performance of RF-based HAR systems [26,27]. The authors of [36] employed a C-band FMCW and a K-band CW radar system in individual and cooperative mode to classify human activities and falls using the SVM classifier. In the cooperative mode, the SVM classifier was able to classify basic human activities with 89% accuracy compared to 70% and 75% accuracies when both radars were used in the individual mode. The solution proposed in this work did not resolve the orientation-independence problem of RF-based HAR systems, because both radars were placed at a 0°aspect angle. However, this work provides a substantial evidence that the fusion of the features extracted from the micro-Doppler signatures of multiple radars improved the overall classification accuracy of the system. In contrast to this previous work [36], our complementary RF sensing approach not only improved the overall classification accuracy but also overcame the orientation-dependency problem of contemporary monostatic radar-based HAR systems.

## 3. System Overview

**Figure 1:**
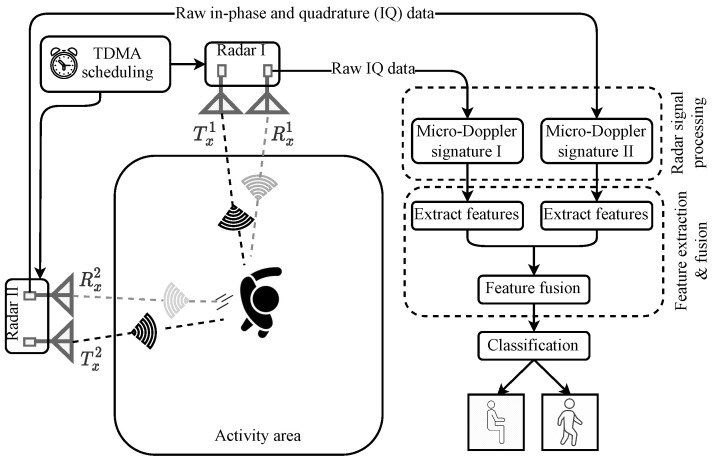
Complementary RF sensing approach for orientation-independent non-invasive HAR and fall detection.

Our approach to designing an orientation-independent non-invasive HAR system mainly comprises of complementary RF sensing and supervised machine learning steps. The complementary RF sensing step uses two monostatic mmWave radar systems, namely: Radar I and Radar II (see Figure 1), which collect RF data in an indoor environment. Both radars are placed orthogonal to each other in the environment to illuminate the target from two different aspect angles, as shown in the system overview diagram.

The radars emit RF signals via their transmission antennas $T_{x}^{i}\;\left(i=1,2\right)$ using TDMA. While propagating in a lossy environment, the transmitted RF signals interact with stationary objects (e.g., the walls and furniture) and non-stationary objects (e.g., a moving person) that are present within the radars’ range. Owing to the Doppler shift phenomenon, the propagating RF signals experience changes in their frequencies as they reflect-off of the moving objects. The receiving antennas $R_{x}^{i}\;\left(i=1,2\right)$ of Radar I and Radar II receive the Doppler-frequency-shifted radar echoes. As we know, the different segments of a person’s body move differently depending on the type of activity. Therefore, each activity leaves behind a distinct Doppler shift pattern. We employ radar signal processing techniques (see Section 4) to compute the micro-Doppler signature from the raw IQ data recorded by each radar. The micro-Doppler signature is sensitive to the aspect angle between the boresight of a radar and the direction of motion of the target. Therefore, a single monostatic radar cannot consistently output unique micro-Doppler signatures for the same activity performed at different aspect angles. Our complementary RF sensing approach overcame this physical limitation of single monostatic radars by combining the micro-Doppler information from two monostatic radars. To achieve this, we first estimate the MDS from the micro-Doppler signatures of Radar I and Radar II. In the machine learning phase, we separately extract statistical and time- and frequency-domain features. Thereafter, feature-level fusion is performed to fuse these features together. Finally, the fused feature set are used to train a supervised machine learning model that could robustly classify orientation-independent human activities.

## 4. Distributed mmWave MIMO Radar System

In this article, we utilized an off-the-shelf K-band FMCW MIMO radar (Ancortek SDR-KIT 2400T2R4) as the RF sensor. Within the context of this paper, we will use the shortened term SDR-KIT to refer to the Ancortek SDR-KIT 2400T2R4. This radar system operates in the 24–26 GHz frequency range and consists of two transmission and four receiving (2×4) RF chains; each transmission and receiving antenna is equipped with an external horn antenna. Thus, the SDR-KIT provides the flexibility to easily distribute $T_{x}$ and $R_{x}$ antennas in indoor environments to illuminate the target (moving person) from two different perspectives. We set up the SDR-KIT in a 2×2 MIMO configuration. Thus, two transmission $T_{x}^{i}\;\left(i=1,2\right)$ and two receiving $R_{x}^{i}\;\left(i=1,2\right)$ antennas were used to transmit and receive RF signals. The $T_{x}^{i}$ and $R_{x}^{i}$ antennas were distributed in pairs in the environment, and each pair of antennas consisted of one collocated $T_{x}$ and $R_{x}$ antenna. This setup allows a single distributed mmWave MIMO radar to be used as two separate monostatic mmWave radar subsystems, i.e., Radar I and Radar II. Both radars emit identical chirp waveforms $s_{T_{x}}\left(t^{\prime }\right)$ as RF signals through their $T_{x}^{i}$ antennas in distinct time slots according to the TDMA scheme. For the two transmitters of the 2×2 MIMO radar system, the time slots are defined as the intervals $\left(2n+i-1\right)T_{sw}\leq t^{\prime }<\left(2n+i\right)T_{sw}$, where n=0,1,… and i=1,2; the symbol $t^{\prime }$ denotes the fast time, and $T_{sw}$ is the chirp’s sweep time. As the signal preprocessing chains of Radar I and Radar II are identical, we will first formulate the expressions corresponding to a single radar. Later in the section, we will provide an expression corresponding to the MDS of the 2×2 MIMO radar system. Within the chirp’s sweep time $T_{sw}$, the transmitter $T_{x}$ emit chirp signals $s_{T_{x}}\left(t^{\prime }\right)$ of the form [37]

(1) $$s_{T_{x}}\left(t^{\prime }\right)=\exp\left[j2\pi \left(f_{0}t^{\prime }+\frac{\gamma }{2}t^{\prime 2}\right)\right],\;\;0\leq t^{\prime }<T_{sw},$$

where $f_{0}$ and γ are the start frequency and chirp slope, respectively. The transmitted signal $s_{T_{x}}\left(t^{\prime }\right)$ reflects back to the radar receiver from stationary and non-stationary scatterers that are present in the environment. For a scatterer with the radar range *R*, the transmitted signal $s_{T_{x}}\left(t^{\prime }\right)$ encounters a propagation delay τ=2R/c, where *c* denotes to the speed of light. Note that the signal reflected from the scatterer underwent an RF mixer stage that downconverted the RF signal to the baseband signal. An ADC then digitized the baseband signal. The baseband signal of an FMCW radar is commonly referred to as the beat signal $s_{b}\left(t^{\prime }\right)$, given as [38]

(2) $$s_{b}\left(t^{\prime }\right)=a\exp\left[j\left(2\pi f_{b}^{\prime }t^{\prime }+\phi \right)\right]$$

where *a*, $f_{b}^{\prime }$, and ϕ are the amplitude, beat frequency, and phase of the baseband signal, respectively. Let the symbol *n* denote the number of fast-time samples in a chirp and *m* denote the number of chirps in the CPI [39,40] of the radar. Then, for the radar’s CPI, the samples of the digitized baseband signal $s_{b}\left(t^{\prime }\right)$ can be arranged in the fast-time $t^{\prime }$ and slow-time *t* directions in the rows and columns of a raw data matrix of order n×m, respectively. Thus, the baseband signal $s_{b}\left(t^{\prime }\right)$ becomes a function of the fast time $t^{\prime }$ and slow time *t*, and will be referred to as $s_{b}\left(t,t^{\prime }\right)$. We could now compute the beat frequency profile $S_{b}\left(f_{b},t\right)$ using the expression [4]

(3) $$\begin{array}{c} S_{b}\left(f_{b},t\right)=\int_{0}^{T_{sw}}s_{b}\left(t,t^{\prime }\right)\exp\left[-j2\pi f_{b}t^{\prime }\right]dt^{\prime }. \end{array}$$

A rectangular window function $W_{r}\left(\cdot \right)$ was then applied to the beat frequency profile $S_{b}\left(f_{b},t\right)$ to obtain the STFT with respect to the slow time *t*, i.e.,

(4) $$\begin{array}{c} X\left(f_{b},f,t\right)=\int_{-\infty }^{\infty }S_{b}\left(f_{b},t^{\prime \prime }\right)W_{r}\left(t^{\prime \prime }-t\right)\exp\left[-j2\pi ft^{\prime \prime }\right]dt^{\prime \prime } \end{array}$$

where *f* and $t^{\prime \prime }$ are the Doppler frequency and running time, respectively. The TV micro-Doppler signature S(f,t) is then obtained as [41]

(5) $$\begin{array}{c} S\left(f,t\right)={\left|\int_{0}^{f_{b,\max}}X\left(f_{b},f,t\right)df_{b}\right|}^{2} \end{array}$$

where $f_{b,\max}$ is the radar’s maximum resolvable beat frequency [42]. For a single radar, we can finally compute the TV MDS $B_{f}^{\left(1\right)}\left(t\right)$ from the TV micro-Doppler signature S(f,t) using

(6) $$\begin{array}{c} B_{f}^{\left(1\right)}\left(t\right)=\frac{\int_{-\infty }^{\infty }fS\left(f,t\right)df}{\int_{-\infty }^{\infty }S\left(f,t\right)df}. \end{array}$$

Similarly, for the 2×2 MIMO radar system, the TV MDS $B_{T_{x}^{i}R_{x}^{i}}^{\left(1\right)}\left(t\right)$ can be obtained as

(7) $$\begin{array}{c} B_{T_{x}^{i}R_{x}^{i}}^{\left(1\right)}\left(t\right)=\frac{\int_{-\infty }^{\infty }fS_{T_{x}^{i}R_{x}^{i}}\left(f,t\right)df}{\int_{-\infty }^{\infty }S_{T_{x}^{i}R_{x}^{i}}\left(f,t\right)df} \end{array}$$

where $S_{T_{x}^{i}R_{x}^{i}}\left(f,t\right)$ is the TV micro-Doppler signature of the wireless link between the transmitter antenna $T_{x}^{i}$ and receiver antenna $R_{x}^{i}$. The TV MDS $B_{T_{x}^{i}R_{x}^{i}}^{\left(1\right)}\left(t\right)$ in (7) is the final and main output of the MIMO radar signal preprocessor from which we derive several features, as described in Section 7.1.

## 5. Experimental Setup and Data Collection

**Figure 2:**
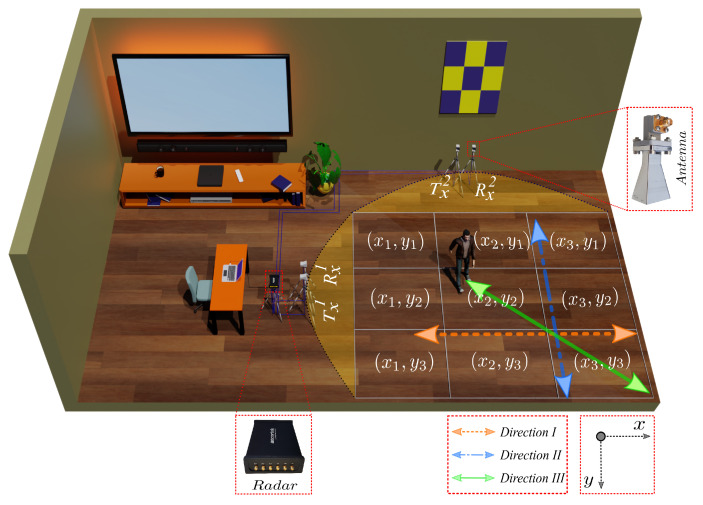
Experimental setup for orientation-independent indoor human activity recognition.

As described in Section 4, the SDR-KIT used in this work was set up to subsist as two monostatic radar subsystems, denoted as Radar I and Radar II, which operated in a TDMA mode. We deployed the SDR-KIT in an indoor environment to collect orientation-independent human activity data. In this case, $T_{x}^{1}$ and $R_{x}^{1}$ horn antennas (see Figure 2) are the transmission and receiving antennas of Radar I, and $T_{x}^{2}$ and $R_{x}^{2}$ are the transmission and receiving antennas of Radar II, respectively. To avoid interference between radar subsystems (Radar I and Radar II), we used RF cables of different lengths for each pair of collocated antennas of Radar I and Radar II [43,44]. A complete list of the system parameters of the SDR-KIT and the lengths of the RF cables used in the experimental setup are provided in Table 1. To illuminate the environment from two different perspectives, we placed the antennas of Radar I and Radar II orthogonal to each other in the environment (see Figure 2). For ease of placement, we mounted the SDR-KIT and the collocated antenna pair of each radar on a separate tripod. All antennas were mounted at a height of 110 cm above the floor. First, we identified the activity region, which was an approximately 4 m^2^ common area covered by the FOV of the $T_{x}^{i}$ and $R_{x}^{i}$ antennas of Radar I and Radar II.

As shown in Figure 2, the activity region was divided into a 3×3 grid. We collected human activity data from six volunteers. The volunteers performed the following five activities: normal walking, sitting on a chair from a standing position, standing up from a chair from a sitting position, picking up an object from the floor from a standing position, and falling onto a mattress placed on the floor from a standing position. As shown in Figure 2, the volunteers repeated each of the above activities multiple times in different grid cells in three different directions denoted as Direction I (facing towards the x-axis), Direction II (facing towards the y-axis), and Direction III (facing along the diagonal line passing through the x-axis and y-axis). The demographics of the volunteers and the number of trials per activity performed by each volunteer are presented in Table 2. We divided the volunteers into two groups based on the extent of their consent. The volunteers of Group A agreed to participate in an extensive data collection campaign, whereas the volunteers of Group B consented to participate in a limited data collection campaign. Furthermore, volunteers 5 and 6 opted out of performing the falling activity due to the associated risk of injuries.

The single grid cell of the activity region was relatively small for performing the walk or fall activities; therefore, these activities spanned multiple grid cells. For example, the volunteers in Group A performed walking activity trials by walking from ($x_{1},y_{1}$) to ($x_{3},y_{3}$) and vice versa; from ($x_{1},y_{1}$) to ($x_{3},y_{1}$) and vice versa; from ($x_{1},y_{1}$) to ($x_{1},y_{3}$) and vice versa; from ($x_{1},y_{2}$) to ($x_{3},y_{2}$); and finally from ($x_{2},y_{1}$) to ($x_{2},y_{3}$) and vice versa. The falling activity trials of the volunteers in Group A were recorded in several directions in different grid cells. For example, we first placed the mattress at the location ($x_{1},y_{2}$), and then the volunteer fell onto the mattress following the direction from ($x_{3},y_{2}$) to ($x_{1},y_{2}$). Similarly, we placed the mattress at locations ($x_{3},y_{2}$); ($x_{2},y_{1}$); ($x_{2},y_{3}$); ($x_{1},y_{1}$); and ($x_{3},y_{3}$). The volunteers fell on the mattress in the following directions: from ($x_{1},y_{2}$) to ($x_{3},y_{2}$); from ($x_{2},y_{3}$) to ($x_{2},y_{1}$); from ($x_{2},y_{1}$) to ($x_{2},y_{3}$); from ($x_{3},y_{3}$) to ($x_{1},y_{1}$); and finally from ($x_{1},y_{1}$) to ($x_{3},y_{3}$), respectively. Finally, the volunteers in Group A performed three more activities: sitting on a chair, standing up from the chair, and picking up an object from the floor. These were repeated in each direction in all grid cells except grid cell ($x_{1},y_{3}$), because the micro-Doppler signature at location ($x_{1},y_{3}$) turned out to be quite similar to the micro-Doppler signature at location ($x_{2},y_{3}$). The volunteers in Group B performed similar walking and falling activities as those in Group A, but they performed fewer trials. The volunteers in Group B performed 3 trials of each of the sitting, standing, and picking up an object activities at only three locations, namely ($x_{1},y_{2}$); ($x_{2},y_{2}$); and ($x_{2},y_{1}$). All in all, we recorded 1398 activity trials of the five activities (walking, falling, sitting, standing, and picking up an object from the floor). The volunteers were told to stay still in their initial pose and wait until they heard a beep indicating that Radar I and Radar II were in the recording mode. Thereafter, the volunteer had to perform the desired activity within 10 s and stay still in their final pose upon completing the activity. The radars were set to automatically stop recording data after 10 s. The recorded raw IQ data of each activity trial were stored in a separate file, which was labeled according to a naming convention for the ease of data management.

## 6. Data Processing

**Figure 3:**
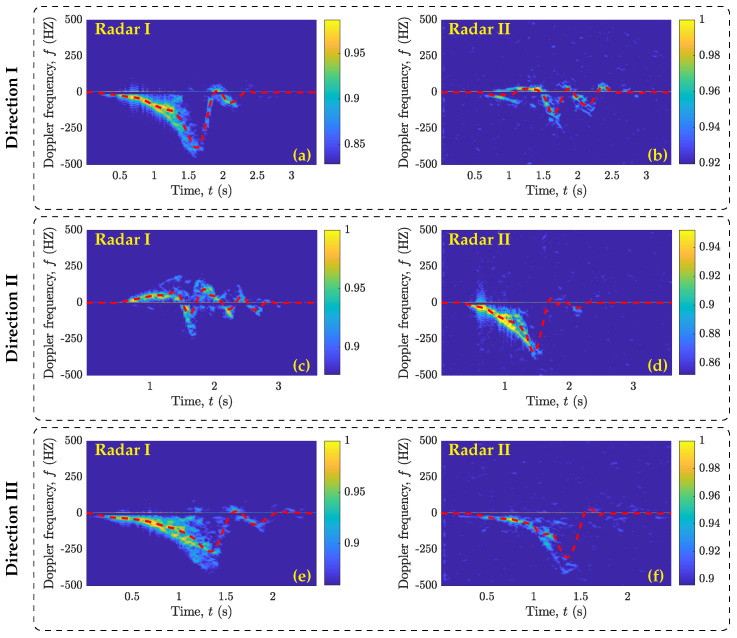
Micro-Doppler signatures (spectrograms) and MDS patterns (red dashed lines) of the falling activity performed in three different directions with respect to Radar I and Radar II as described in Section 5 and shown in Figure 2.

**Figure 4:**
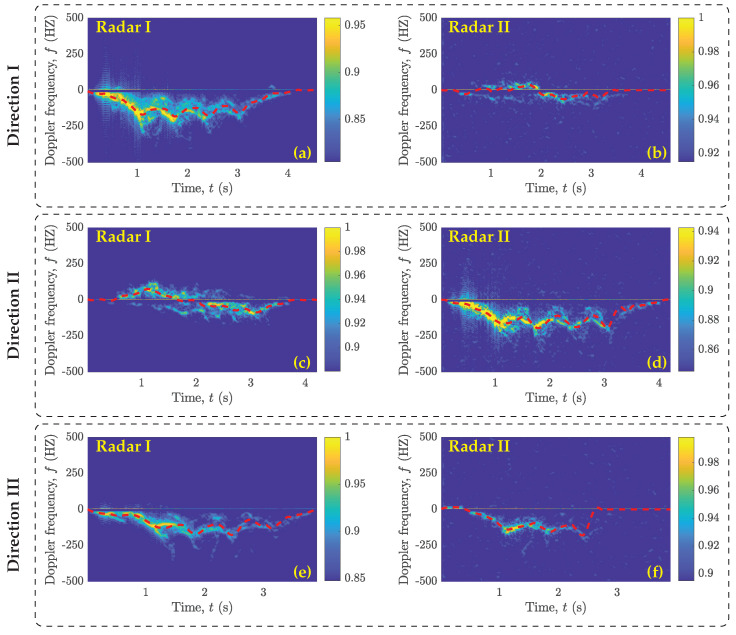
Micro-Doppler signatures (spectrograms) and MDS patterns (red dashed lines) of the walking activity performed in three different directions with respect to Radar I and Radar II as described in Section 5 and shown Figure 2.

As described earlier in Section 5, each activity trial in our dataset was 10 s long, including active and idle time periods. In this work, an active time period is considered as a 2.5–5 s interval during which a volunteer performed an activity. The duration of an active time period differed across activity trials, because it depended on the type of activity and the speed at which the volunteer performed the activity. In contrast, the time periods during which the volunteer stood still are defined as idle time periods. In each activity trial of our dataset, the idle time periods occurred before and after the activity time period. We used the variance-based thresholding method (referred to herein as VTM) [45] to automatically determine the start and end of an active time period in the recorded raw IQ data. The VTM takes in the high-pass filtered in-phase component of the raw IQ data. Then, the VTM computes the moving variance of the input data and compares the variance values with a predefined threshold level. The VTM marks the start and end of an active time period when the variance rises above and drops below a threshold level, respectively. Once all active time periods are marked, we chose the one that is larger than 1.25 s. This allowed the VTM to prune the small segments that might have resulted from noise or small movements of body segments during the idle time periods. Finally, the start and end time stamps of the identified active time period are used to segment the activity from the raw IQ data. It is important to note that the segmented raw activity data are still in the form of the complex baseband domain. The segmented raw activity data are then passed to the radar signal processing module (see Section 4), which first rearranges the beat signal $s_{b}\left(t^{\prime }\right)$ with respect to the slow time *t* and fast time $t^{\prime }$, producing $s_{b}\left(t,t^{\prime }\right)$, and then computes the beat frequency profile $S_{b}\left(f_{b},t\right)$ according to (3), followed by the micro-Doppler signature S(f,t) introduced in (5) and the MDS $B_{f}^{\left(1\right)}\left(t\right)$ as defined in (6). The micro-Doppler signatures and the MDS patterns of the falling and walking activities are presented in Figure 3 and Figure 4.

**Figure A1:**
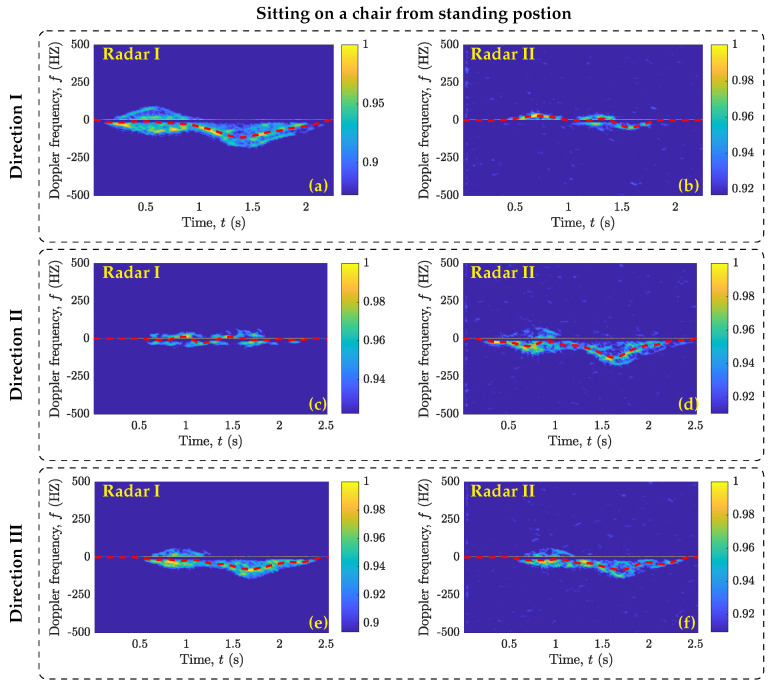
Micro-Doppler signatures (spectrograms) and MDS patterns (red dashed lines) of the sitting activity performed in three different directions with respect to Radar I and Radar II (see Section 5 and Figure 2).

**Figure A2:**
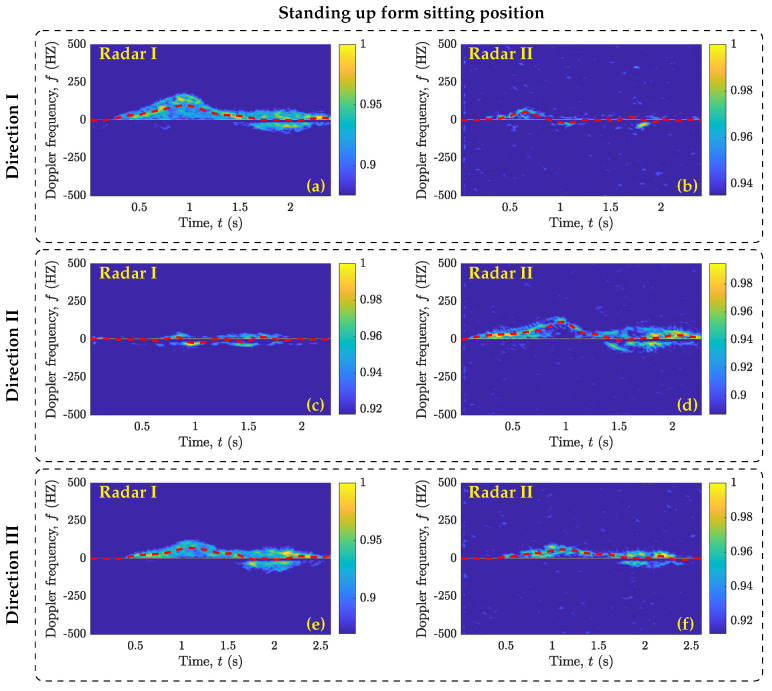
Micro-Doppler signatures (spectrograms) and MDS patterns (red dashed lines) of the standing up from a chair activity performed in three different directions with respect to Radar I and Radar II (see Section 5 and Figure 2).

**Figure A3:**
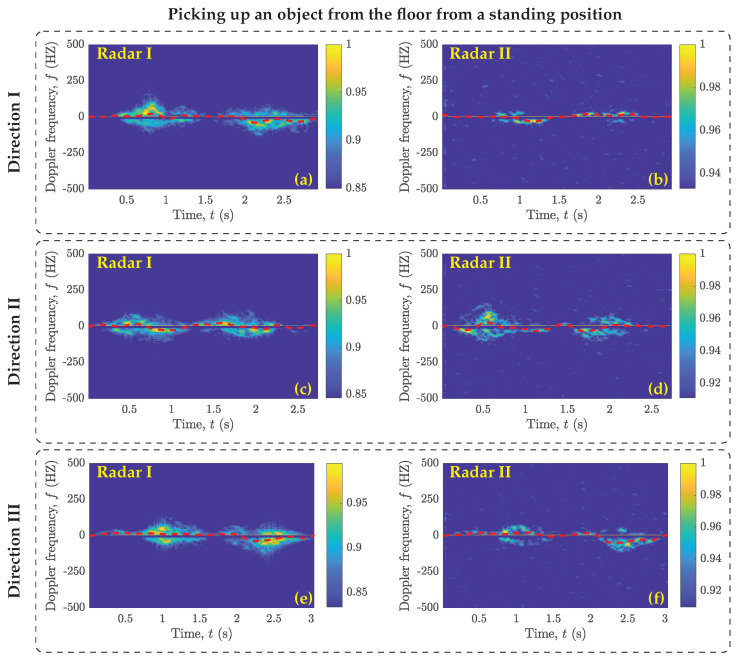
Micro-Doppler signatures (spectrograms) and MDS patterns (red dashed lines) of the picking up an object from the floor from a standing position activity performed in three different directions with respect to Radar I and Radar II (see Section 5 and Figure 2).

To keep this section concise and for the completeness of the article, the micro-Doppler signatures and the MDS patterns of the activities of sitting on a chair, standing up from a chair, and picking up an object from the floor are provided in Appendix B (see Figure A1, Figure A2 and Figure A3). We can observe that the MDS patterns (or micro-Doppler signatures) produced by Radar I and Radar II for the falling activity performed in Direction I from location ($x_{1},y_{2}$) to ($x_{3},y_{2}$) were significantly different from each other (see Figure 3a,b). Since Radar I and Radar II were placed orthogonal to each other in our experimental setup, the trajectory of the falling activity performed in Direction I was along a 0° aspect angle with respect to Radar I and along a 90° angle with respect to Radar II. Similarly, the falling activity performed in Direction II from location $\left(x_{2},y_{1}\right)$ to $\left(x_{2},y_{3}\right)$ was seen at a 0° aspect angle by Radar II and a 90° aspect angle by Radar I (see Figure 3c,d). Therefore, the MDS patterns in Figure 3a,d and in Figure 3b,c appeared similar to each other. In Direction III (i.e., from location $\left(x_{1},y_{1}\right)$ to $\left(x_{3},y_{3}\right)$), the trajectory of the falling activity makes approximately similar aspect angles with Radar I and Radar II. Thus, the MDS patterns produced by both radars turned out to be quite similar to each other. Furthermore, by comparing only the outputs of Radar I (see Figure 3a,c,e) or Radar II (see Figure 3b,d,f), we can observe how the micro-Doppler signature of an activity deteriorated as the aspect angle changed from 0° (Direction I) through 45° (Direction III) to 90° (Direction II). Similar observations can also be noted from the MDS patterns of the walking (see Figure 4a–f), sitting (see Figure A1a–f), standing (see Figure A2a–f), and picking (see Figure A3a–f) activities. The data processing modules processed each recorded activity trial. While processing the data, we found that 34 activity trials resulted into incomplete MDS patterns or micro-Doppler signatures. This occurred in situations where the volunteers either performed an activity trial before hearing the beep or could not keep track of time after hearing the beep and performed the activity trial in the last moments of the 10 s activity recording interval. We discarded such incomplete activity trials. From each of the remaining 1364 activity trials, we extracted the MDS patterns corresponding to Radar I and Radar II.

## 7. Classifying Human Activities

Within the context of this work, we used the supervised learning setup to classify human activities into the following K=5 classes: walking, falling, sitting, standing, and picking up an object from the floor. To achieve this, first, we prepared the labeled dataset D=${\left\{\left(x_{i},y_{i}\right)|x_{i}\in R^{P},y_{i}\in \left\{1,2,\ldots ,K\right\}\right\}}_{i=1}^{N}$ consisting of *N* elements, where *N* is the total number of processed activity trials. We denote as $x_{i}=\left(x_{i}^{1},x_{i}^{2},\ldots ,x_{i}^{P}\right)$ the P-dimensional feature vector that was extracted from the *i*th processed activity trial, and $y_{i}$ is the label (or the class) of that activity trial.

### 7.1. Feature Extraction and Fusion

As described in Section 5, every volunteer performed several trials of each activity. We processed each activity trial as described in Section 6, which resulted in MDS pattern corresponding to Radar I and Radar II. These MDS patterns represent the average Doppler signature of the performed activity trial with respect to Radar I and Radar II. We extracted a total of 40 statistical and time- and frequency-domain features separately from the MDS pattern corresponding to each radar. The explanation of these features is provided in Table A1 of Appendix A. We used these feature vectors to prepare three labelled feature sets $D_{1}$, $D_{2}$, and $D_{3}$. We refer to these feature sets as the Radar I, Radar II, and complementary feature sets. The Radar I feature set contained only those feature vectors that were extracted from the MDS patterns corresponding to Radar I. Similarly, the Radar II feature set contained only the feature vectors that were extracted from the MDS patterns corresponding to Radar II. We labelled each feature vector of the Radar I and Radar II feature sets according to the type of activity. Finally, the complementary feature set was obtained by serially concatenating the feature vectors of the same activity trials of the Radar I and Radar II feature sets.

### 7.2. Classification Using SVM

In this work, we used an SVM [46] to classify human activities. An SVM is a supervised learning model that classifies data into positive and negative classes. In this work, the terms classifier and model are used interchangeably. To achieve this, an SVM classifier uses labeled training data $D_{TR}={\left\{\left(x_{i},y_{i}\right)|x_{i}\in R^{P},y_{i}\in \left\{+1,-1\right\}\right\}}_{i=1}^{N}$ to find an optimal high-dimensional hyperplane H that has a maximum distance (or margin) to the nearest data points of both classes. The hyperplane H is parameterized by a weight vector $w\in R^{P}$ and a bias b∈R such that each training example $x_{i}$ in $D_{TR}$ can be described by $y_{i}\left(w^{T}x_{i}+b\right)\geq 1-\xi_{i}$. Here, ${\left(\cdot \right)}^{T}$ denotes the transpose operator, and the symbol $\xi_{i}\geq 0$ is called the slack variable, which penalizes those training samples that are either misclassified or fall within the margin boundary. This facilitates the construction of a margin that allows for some misclassifications. To construct such a maximum margin hyperplane H, the SVM finds the weight vector w and the bias *b* by solving the following convex minimization problem:

(8) $$\begin{array}{c} \min_{w,b,\xi }\frac{1}{2}w^{T}w+C\sum_{i=1}^{N}\xi_{i}\;s.t.\;y_{i}\left(w^{T}x_{i}+b\right)+\xi_{i}-1\geq 0,\;\xi_{i}\geq 0\;\forall_{i} \end{array}$$

where the symbol *C* denotes a regularization constant that controls the strength of the penalty. The method of Lagrange multipliers was used to cater to the constraints of the objective function presented in (8), which led to the following Lagrangian function:

(9) $$\begin{array}{cc} L= & \frac{1}{2}w^{T}w+C\sum_{i=1}^{N}\xi_{i}-\sum_{i=1}^{N}\alpha_{i}\left(y_{i}\left(w^{T}x_{i}+b\right)+\xi_{i}-1\right)-\sum_{i=1}^{N}\beta_{i}\xi_{i}. \end{array}$$

We transformed (9) into a dual Lagrangian formulation. This could be achieved by differentiating (9) with respect to w, *b*, and $\xi_{i}$ and then equating the derivatives with zeros, resulting in $w=\sum_{i=1}^{N}\alpha_{i}y_{i}x_{i}$, $\sum_{i=1}^{N}\alpha_{i}y_{i}=0$, and $C=\alpha_{i}+\beta_{i}$. Back-substituting these results in (9) produced a dual Lagrangian formulation $L_{D}$ that depended on α, which we aimed to maximize:

(10) $$\begin{array}{cc} L_{D}= & \sum_{i=1}^{N}\alpha_{i}-\frac{1}{2}\sum_{i=1}^{N}\sum_{j=1}^{N}\alpha_{i}\alpha_{j}y_{i}y_{j}K(x{}_{i},x_{j})\;s.t.\;0\leq \alpha_{i}\leq C\;\forall_{i},\;\sum_{i=1}^{N}\alpha_{i}y_{i}=0. \end{array}$$

The dual Lagrangian formulation $L_{D}$ is a QP problem. A QP solver returned α, which was used to compute w and *b* following the KKT conditions. This SVM model could be trained on linearly inseparable training data. However, the performance of the SVM model could be further improved using the kernel trick $K(x{}_{i},x_{j})$, as presented in (10), which simply mapped the low-dimensional feature space onto a high-dimensional feature space. In this way, the non-linear relationships between the features apparently became quite linear [47]. Finally, the trained SVM model could be used to predict the label (or class) $y_{pred}$ of a previously unseen example $x_{\mathrm{Test}}$ as follows:

(11) $$\begin{array}{c} y_{pred}=\mathrm{sign}\left(w^{T}x_{\mathrm{Test}}+b\right). \end{array}$$

### 7.3. Training and Testing the Classifier

Recall that we extracted Radar I, Radar II, and complementary feature sets from the human activity data (see Section 7.1). Each feature set $D_{i}$ was used to train and test a multi-class svm classifier. Before training and testing the classifier, each feature set $D_{i}$ was normalized first and then partitioned into training and test sets. The training set consisted of labelled feature vectors that were used to train the classifier. Once the classifier was trained, the test set was used to assess how accurately the trained classifier could predict the classes of the unseen feature vectors of the test set. We used two different approaches to split each feature set $D_{i}$. In the first approach, we randomly split each feature set $D_{i}$ into a training set $D_{i,Train}$ and a test set $D_{i,Test}$, such that $D_{i,Train}\cap D_{i,Test}=\emptyset$ and $D_{i,Train}\cup D_{i,Test}=D_{i}$, where ∅ denotes an empty set. Moreover, the $D_{i,Train}$ and $D_{i,Test}$ sets consisted of 70% and 30% of the samples of the $D_{i}$ feature set, respectively. In this case, the $D_{i,Train}$ set was used to train an SVM classifier and the $D_{i,Test}$ set was used to evaluate the performance of the trained classifier. Conversely, in the second approach, each feature set $D_{i}$ was divided into $D_{i,A}$ and $D_{i,B}$ subsets according to the groups A and B as described in Table 2, such that $D_{i,A}\cap D_{i,B}\;=\emptyset$ and $D_{i,A}\cap D_{i,B}\;=D_{i}$. The group-wise division of feature sets ensured that the feature vectors in the training and test sets strictly belonged to different users. In this case, we used $D_{i,A}$ as a training set to train an SVM classifier and $D_{i,B}$ as a test set to evaluate the performance of the classifier.

Given a pair of training and test sets, a five-fold gridsearchCV technique was applied to train the SVM classifiers. With gridsearchCV, we aimed to find the optimal parameters of the classifier (i.e., the regularization constant *C*, the type of kernel function, and the kernel parameters) that resulted in the best cross-validation accuracy of the classifier. As is known, the SVM is limited by default to binary-class problems, where the data can be classified into two classes, i.e., K=2. However, in this work, we were interested in classifying human activities into five classes. Therefore, we used the OvO training strategy that enables binary classifiers to cater to multi-class-problems, where K>2. The OvO approach [48] trains K(K−1)/2 binary classifiers in the training phase, each for every possible pair of classes. In the testing phase, each trained classifier predicts the class of every example of the test set, and the class predicted by the majority of classifiers is considered the final class of that test example. We report the performance of each classifier on a test set in terms of the following performance metrics:

(12) $$\begin{array}{cc} \mathrm{Recall} & =\frac{T_{P}}{T_{P}+F_{N}}\times 100\% \end{array}$$

(13) $$\begin{array}{cc} \mathrm{Precision} & =\frac{T_{P}}{T_{P}+F_{P}}\times 100\% \end{array}$$

(14) $$\begin{array}{cc} \mathrm{Accuracy} & =\frac{T_{N}+T_{P}}{T_{N}+F_{N}+T_{P}+F_{P}}\times 100\%. \end{array}$$

In (12)–(14), the terms $T_{P},F_{P},T_{N},$ and $F_{N}$ stand for true positive, false positive, true negative, and false negative, respectively.

### 7.4. Results and Discussion

Remember that there were three feature sets, i.e., Radar I, Radar II, and complementary. We used each of these feature sets to train and evaluate an SVM classifier. For simplicity and consistency, we named the SVM classifiers after the feature groups. For example, the SVM classifier trained and evaluated using the training and test subsets of the Radar I feature set was named the Radar I classifier. By analogy, we named the other two classifiers the Radar II classifier and the complementary classifier. The performances of each classifier are visually described in the confusion matrices below, organized such that the first five columns and first five rows indicate the actual and predicted classes of the test samples in the test set. The last column and the last row of the confusion matrices provide the precision and recall of each class, respectively. Moreover, the diagonal entries shown in green represent the counts of correctly classified test samples (i.e., $T_{P}$ and $T_{N}$) with respect to each class. Conversely, the off-diagonal entries in red provide the counts of misclassified test samples (i.e., $F_{P}$ and $F_{N}$). Finally, the overall accuracy of the classifier and the total number of test samples are provided in the orange cells.

#### 7.4.1. Results of the Radar I and Radar II Classifiers

**Figure 5:**
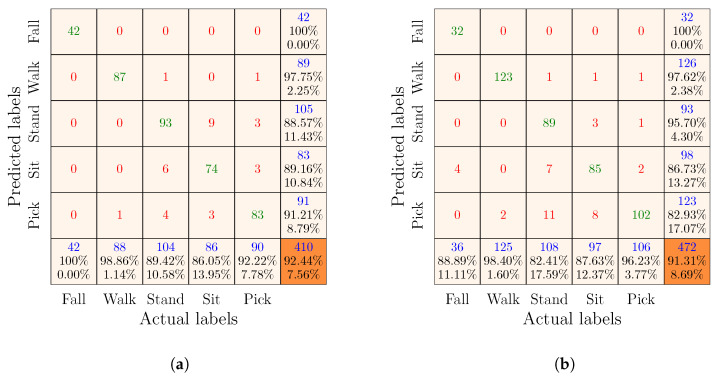
Confusion matrices of the Radar I classifier obtained by the (**a**) random and (**b**) group-wise splitting of the Radar I feature set.

**Figure 6:**
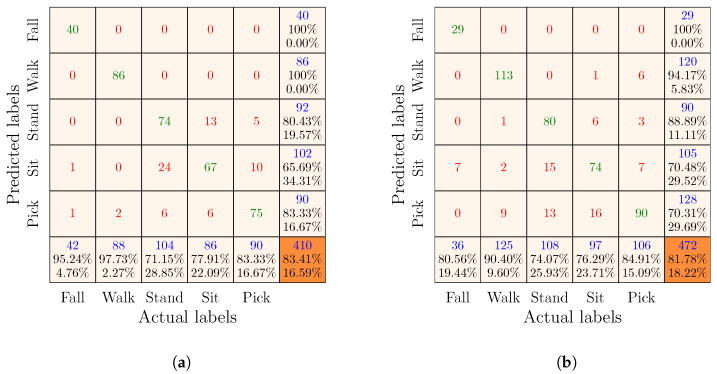
Confusion matrices of the Radar II classifier obtained by the (**a**) random and (**b**) group-wise splitting of the Radar II feature set.

The results of the Radar I and Radar II classifiers are presented in Figure 5 and Figure 6, respectively. Individually Radar I or Radar II represents a scenario in which a person performs activities in different orientations in front of only a single monostatic radar that was placed in the environment. In Figure 5a, we can observe that the overall recognition accuracy of the Radar I classifier was 92.44% when the training and test sets were generated by randomly splitting the Radar I feature set. Whereas, the overall recognition accuracy of the Radar I classifier decreased to 91.31% when it was trained and evaluated with the training and test sets obtained by the group-wise partitioning of the Radar I feature set. This slight degradation in overall accuracy was to be expected owing to the fact that the group-wise splitting ensured that training and test sets strictly consisted of data from different volunteers, thus providing a highly realistic evaluation of the classifier. Moreover, we can notice that the Radar I classifier was able to classify each activity with reasonable precision and recall, irrespective of the strategy used to split the Radar I feature vectors into training and test sets. However, comparing the results of the Radar I classifier with other existing works on mmWave radar-based HAR [15,20,21,22,23,24,25], it can be argued that the recognition accuracy of the Radar I classifier is at the lower end. This is due to the fact that the Radar I classifier attempted to recognize orientation-independent activities, unlike other approaches that recognized activities performed along the 0° aspect angle.

Presumably, one could argue that the Radar I and Radar II classifiers should have reported similar classification results. However, this is not the case when we compare the confusion matrices in Figure 5a and Figure 6a or in Figure 5b and Figure 6b. We find that the classification accuracy of the Radar II classifier is approximately 10% lower than that of the Radar I classifier. This problem is related to the SDR-KIT that we used in this work. The SDR-KIT suffers from the problem of inter-channel interference. To reduce the effect of inter-channel interference, we opted for the solution proposed in [43,44], which required the use of RF delay lines for different subchannels. As proposed in [43,44], we used RF cables of different lengths for Radar I and Radar II to connect the $T_{x}^{i}$ and $R_{x}^{i}$ antennas. As shown in Table 1, the $T_{x}^{2}$ and $R_{x}^{2}$ antennas of Radar II were connected to the SDR-KIT using 7 m long RF cables, and each of these RF cables were constructed by splicing together three 2 m and one 1 m long RF cables. Consequently, this attenuated the power of the transmitted RF signal of Radar II. Therefore, the micro-Doppler signatures and the MDS patterns computed using the RF signals received by the Radar II were nosier (see Figure 3, Figure 4, Figure A1, Figure A2, and Figure A3), resulting in the lower classification accuracy of the Radar II classifier.

#### 7.4.2. Results of the Complementary Classifier

**Figure 7:**
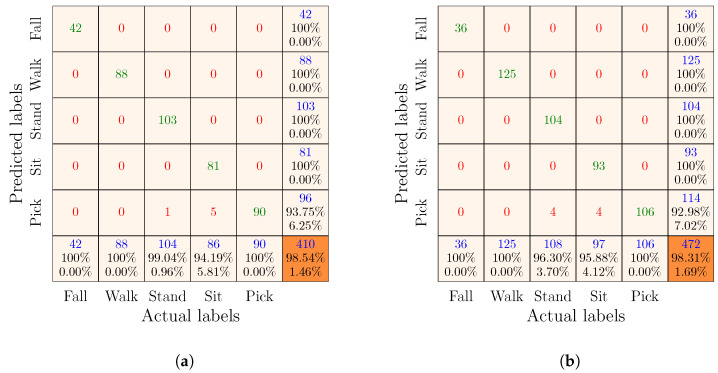
Confusion matrices of the complementary classifier obtained by the (**a**) random and (**b**) group-wise partitioning of the complementary feature set.

The results of the complementary classifier are presented in Figure 7. Note that the complementary classifier was trained and evaluated using the complementary feature set, which was obtained by fusing the Radar I and Radar II feature sets. In Figure 7a, we can observe that the complementary classifier could classify orientation-independent human activities with 98.54% accuracy using the training and test sets that were obtained by randomly splitting the complementary feature set. Whereas, the complementary classifier achieved 98.31% classification accuracy when it was trained and tested using the group-wise partitioned training and test sets (see Figure 7b). In addition, the complementary classifier predicted fall, walk, sit, and stand activities with 100% precision. On the other hand, the recall for each of the fall, walk, and pick activities was 100%. Evidently, the complementary classifier made significantly fewer classification errors. For instance, the complementary classifier misclassified only eight test samples (see Figure 7b) compared to the 41 and 86 classifications errors made by the Radar I (see Figure 5b) and Radar II (see Figure 6b) classifiers, respectively. Four of these eight misclassified test samples belonged to the standing activity and the remaining four to the sitting activity. Thus, the precision of the pick activity was 93%, and the recall of the stand and sit activities was 96.30% and 98.88%, respectively. A comparison of the results of the complementary (see Figure 7b), Radar I, and Radar II classifiers (see Figure 5b and Figure 6b, respectively) provides a strong evidence that using multiple monostatic radars to illuminate an environment from multiple perspectives not only improves the overall classification accuracy but also allows orientation-independent human activities to be recognized with high accuracy. Furthermore, by comparing these results with those of related works (see Section 2), we argue that our complementary RF sensing-based HAR approach outperformed previous methods [15,20,21,22,25] in terms of overall classification accuracy.

The proposed complementary RF sensing approach is flexible and adaptable so that it can be scaled to meet the complexity of orientation-independent activities and gesture recognition tasks by adding more radars. In addition, this complementary RF sensing approach will serve as the basis for developing orientation-independent multi-person HAR systems. However, it should be noted that the complementary RF sensing technique for HAR is computationally more expensive compared to the existing RF-based HAR approaches.

## 8. Conclusions

The existing radar-based HAR systems in the literature generally employ a single monostatic radar to recognize human activities. These existing solutions work very well in recognizing human activities performed along the boresight (0° aspect angle) of the radar. However, their recognition accuracy gradually starts decreasing as the aspect angle between the radar and the target’s direction of motion increases. Therefore, the existing solutions generally struggle to recognize orientation-independent human activities. This is because activities performed at different aspect angles in front of a single monostatic radar produce different micro-Doppler signatures. In this article, we proposed a complementary RF sensing approach to resolve the limitations of contemporary radar-based HAR systems and recognize orientation-independent human activities. Our approach mainly consisted of RF sensing and machine learning phases. In the RF sensing phase, we used an Ancortek SDR-KIT 2400T2R4, which was configured as two monostatic radars that were placed orthogonal to each other to illuminate the target (i.e., moving person) from two different aspect angles. We used this setup to collect an orientation-independent dataset from six volunteers. We collectively recorded more than 1350 trials of five different activities that were performed in different orientations and locations within the FOV of both radars. We processed the raw IQ data of both radars to obtain the micro-Doppler signatures. Subsequently, we used the micro-Doppler signatures to compute the MDS patterns. In the machine learning phase, we first extracted various features from the MDS patterns of both radars and then fused them together. We organized these features into three sets, namely the Radar I, Radar II, and complementary feature sets. We used each feature set to train and test an SVM classifier in random and group-wise split scenarios. Our results showed that the complementary SVM classifier achieved overall classification accuracies of 98.54% and 98.31% in random and group-wise split scenarios, respectively, whereas the Radar I classifier achieved classification accuracies of 92.44% and 91.31% and the Radar II classifier achieved classification accuracies of 83.41% and 81.78% in random and group-wise split scenarios, respectively. Despite the fact that Radar II underperformed in our experiments due to hardware-related technical challenges, the two radars complemented each other well enough that the classifier trained and evaluated using their combined bi-perspective information (i.e., the complementary feature set) outperformed the classifiers that were trained and evaluated using an individual radar’s uni-perspective information (the Radar I and Radar II feature sets). Our experimental results and the scalability of the proposed system suggest that complementary RF sensing has significant potential for realizing highly accurate, non-invasive, user-friendly, and privacy-preserving complex HAR systems. In future work, we will employ different feature selection methods (e.g., information gain, chi-square test, forward feature selection, and backwards and recursive feature elimination) to further improve the performance of proposed complementary RF-based HAR system. In addition we will also compare the performance of different machine and deep learning algorithms such as decision trees, random forests, deep CNN, and LSTM models. 

## Figures and Tables

**Table 1:** Radar system parameters for the experimental setup.

Parameter	Symbol	Value
Carrier frequency	$f_{c}$	24.125 GHz
Bandwidth	*B*	250 MHz
Sweep time	$T_{sw}$	500 µs
Pulse repetition frequency	PRF	1 KHz
RF cable lengths (Radar I)	$\left(T_{x}^{1},R_{x}^{1}\right)$	(0.3,0.3) m
RF cable lengths (Radar II)	$\left(T_{x}^{2},R_{x}^{2}\right)$	(7.0,7.0) m

**Table 2:** Demographics of volunteers and tabular representation of the collected data.

Group	Volunteer	Gender (Male/Female)	Age (Years)	Activity Trials				
				Fall	Walk	Stand	Sit	Pick
A	1	m	35	60	80	105	105	105
	2	m	34	60	80	105	105	105
B	3	m	27	18	40	27	27	27
	4	m	36	18	40	27	27	27
	5	f	33	–	24	27	27	27
	6	m	30	–	24	27	27	27

## Data Availability

The processed data that support the findings of this study are available from the corresponding author on reasonable request.

## Abbreviations

The following abbreviations are used in this manuscript:

FMCW	frequency-modulated continuous wave
CPI	coherent processing interval
TV	time-variant
ADC	analog-to-digital converter
FM	frequency-modulated
WHO	World Health Organization
FFT	fast Fourier transform
FD	fall detection
SISO	single-input signle-output
MIMO	multiple-input multiple-output
HAR	human activity recognition
AAL	active assisted living
CTF	channel transfer function
CSI	channel state information
IMU	inertial measurement unit
RSSI	received signal strength indicator
RF	radio frequency
MDS	mean Doppler shift
NIC	network interface card
SVM	support vector machine
SIMO	single-input multiple-output
CFO	carrier frequency offset
SFO	sampling frequency offset
PCA	principal component analysis
RMS	root mean square
OFDM	orthogonal frequency-division multiplexing
STFT	short-time Fourier transform
LOESS	locally estimated scatterplot smoothing
LOSO	leave-one-subject-out
VTM	variance-based thresholding method
MAV	mean absolute value
EMAV	enhanced mean absolute value
EWL	enhanced waveform length
WMAVI	weighted mean absolute value I
WMAVII	weighted mean absolute value II
MAC	mean amplitude change
DASDV	difference absolute standard deviation
SSI	simple squared integral
SSC	slope sign change
MFL	maximum fractal length
OvO	one-versus-one
OvA	one-versus-all
ADL	activities of daily living
CNN	convolutional neural network
TUG	timed up and go
CGA	comprehensive geriatric assessment
WLPCA	widely linear PCA
WLCKPCA	widely linear complex kernel PCA
mmWave	millimeter-wave
SDR	software-defined radio
FOV	field of view
KKT	Karush–Kuhn–Tucker
QP	quadratic programming
GridsearchCV	grid search cross-validation
TDMA	time division multiple access
IQ	in-phase and quadrature
LSTM	long short-term memory
CW	continuous wave

## Appendix A. A Description of Features Extracted from Mean Doppler Shift Patterns

**Table A1:** A description of features extracted from the MDS pattern.

Feature	Description
Mean	Computes the sample mean of the MDS.
Median	Computes the median of the MDS.
Variance	Computes the variance of the MDS.
Skewness	Computes the asymmetric spread of the MDS about its mean.
Kurtosis	Computes the fourth standardized moment of the MDS.
Minimum value	Returns the minimum value of the MDS.
Maximum value	Returns the maximum value of the MDS.
Inter-quartile range	Measures the dispersion of the MDS by computing the difference in the 75th and 25th percentiles of the MDS.
Mean absolute deviation	Measures the variability in the MDS by computing the average distance between each sample in the MDS and the sample mean of the MDS.
Slope	Computes the slope of the MDS by fitting a linear equation to the samples of the MDS.
Entropy	Computes the randomness of the MDS using Shanon entropy.
Total energy	Computes the power of the MDS at a given time.
Signal distance	Computes the distance traveled by the MDS using the hypotenuse between two samples of the MDS.
Mean difference	Computes the mean of the differences in samples of the MDS.
Absolute energy	Computes the absolute energy of the MDS.
Temporal centroid	Computes the center of gravity of the energy envelop of the MDS.
Median difference	Computes the median of the differences in samples of the MDS.
Zero crossing rate	Computes the rate at which the MDS crosses the zero axis.
Area under the curve	Computes the area under the curve of the MDS using the trapezoid rule.
Peak-to-peak distance	Computes the absolute difference between the maximum and minimum values of the MDS.
Negative turning points	Computes the number of negative turning points in the MDS.
Positive turning points	Computes the number of positive turning points in the MDS.
Mean absolute difference	Computes the mean of the absolute differences in samples of the MDS.
Sum of absolute difference	Computes the sum of the absolute differences in samples of the MDS.
Median absolute difference	Computes the median of the differences in samples of the MDS.
Spectral slope	Computes the decrease in the spectral amplitude of the MDS.
Spectral spread	Computes the spectral standard deviation of the MDS around its spectral centroid.
Spectral roll-off	Computes the spectral roll-off frequency point of the MDS, where 95% of its magnitude is contained below this frequency point.
Spectral entropy	Computes the spectral power distribution of the MDS.
Spectral distance	Computes the distance of the cumulative sum of the FFT components of the MDS with respective linear regression.
Spectral centroid	Computes the "center of gravity" of the MDS spectrum.
Spectral decrease	Computes the decrease in the spectral amplitude of the MDS, focusing on lower frequencies.
Spectral kurtosis	Computes the flatness of the spectrum of the MDS around its mean value.
Spectral variance	Computes how quickly the power spectrum of the MDS varies.
Power bandwidth	Computes the width of the frequency band in which 95% of the power of the MDS is located.
Spectral skewness	Computes the spectral asymmetry of the MDS around its mean value.
Fundamental frequency	Computes the fundamental frequency of the MDS.
Maximum power spectrum	Computes the maximum value of the power spectrum density of the MDS.
Spectral positive turning points	Computes the number of positive turning points in the magnitude of the FFT of the MDS.

## Appendix B. Activity Signatures

This appendix provides the micro-Doppler signatures for the activities of sitting on a chair, standing up from a chair, and picking up an object from the floor.
